# Patient Portal Activation Disparities by Ward, Census Tract, and Zip Code in an Urban Landscape Among Neurology Patients: Cross-Sectional Multi-Scale Analysis

**DOI:** 10.2196/98695

**Published:** 2026-08-26

**Authors:** Nicholas Streicher

**Affiliations:** 1 Department of Neurology, Georgetown University Medical Center, 3800 Reservoir Road, N.W., Washington, DC, 20007, United States, 1 202 687 0100

**Keywords:** patient portals, digital divide, health equity, health care disparities, neurology, geographic information systems, electronic health records, socioeconomic factors, broadband, health informatics

## Abstract

**Background:**

Patient portals are essential infrastructure, reinforced by the 21st Century Cures Act, yet adoption remains inequitable. The COVID-19 pandemic accelerated portal adoption as telehealth and remote result delivery made electronic access integral to care, but racial and ethnic disparities persisted. Understanding activation determinants is critical for addressing digital health disparities, particularly among neurology patients, for whom cognitive, speech, and mobility impairments can complicate portal use.

**Objective:**

We examined the demographic, geographic, and neighborhood-level factors associated with patient portal activation among neurology patients in the Washington, DC (officially the District of Columbia), metropolitan area.

**Methods:**

We conducted a cross-sectional study of 72,417 patients with at least one outpatient neurology encounter (including telehealth) at 2 academic medical centers sharing a common electronic health record in Washington, DC. The primary outcome was portal activation, defined as having logged into the portal at least once. We examined associations using multivariable logistic regression (reporting adjusted odds ratios [aORs]) adjusting for age, sex, race and ethnicity, visit counts, and year of most recent encounter, and we assessed geographic patterning at multiple scales (the DC metropolitan catchment area, DC’s 8 wards, census tracts via geocoded addresses, and residential zip codes) using Pearson and Spearman correlations between ward- and tract-level American Community Survey indicators and activation.

**Results:**

Portal activation was 64.7% (46,851/72,417) overall; patients averaged 9.7 (SD 18.9) visits. Activation varied by race and ethnicity (non-Hispanic White: 21,420/28,154, 76.1%; non-Hispanic Asian: 1109/1925, 57.6%; non-Hispanic Black: 13,057/22,900, 57%; Hispanic: 1979/3600, 55%). In adjusted models, odds of activation were lower for non-Hispanic Black (aOR 0.46, 95% CI 0.44‐0.48), Hispanic (aOR 0.34, 95% CI 0.31‐0.37), and non-Hispanic Asian (aOR 0.47, 95% CI 0.42‐0.52) patients vs non-Hispanic White patients, and each SD increase in age was associated with lower odds (aOR 0.60, 95% CI 0.59‐0.61; *P*<.001 in all cases). Activation differed across DC wards, from 48% (1023/2131; ward 7) to 82% (1554/1896; ward 2). Ward-level activation correlated strongly with educational attainment (*r*=0.95; *P*<.001), broadband access (*r*=0.89; *P*=.003), and median income (*r*=0.81; *P*=.01); educational attainment was the strongest independent neighborhood-level predictor in joint models. Within individual wards, non-Hispanic White patients activated at 85.7% (1551/1809) to 91.2% (714/783) vs 50.5% (1139/2255) to 63.6% (206/324) for non-Hispanic Black patients, and disparities persisted in a sensitivity analysis restricted to encounters in 2024 to 2026 (non-Hispanic Black patients: aOR 0.37, 95% CI 0.35‐0.39).

**Conclusions:**

To our knowledge, this is the first multi-scale geographic analysis of patient portal activation. Activation was shaped by demographic, socioeconomic, and geographic factors, yet racial disparities persisted within individual wards regardless of socioeconomic advantage, indicating that neighborhood resources alone do not explain the digital divide. Health systems should pair targeted measures such as neighborhood-level enrollment support and digital literacy assistance with culturally tailored, clinic-based activation support to achieve digital health equity.

## Introduction

Patient portals have become central to care delivery, initially driven by the Health Information Technology for Economic and Clinical Health Act of 2009 and more recently advanced by the 21st Century Cures Act, which in 2021 mandated real-time electronic access to test results and accelerated the shift toward portal-based patient-clinician communication [1]. The COVID-19 pandemic acted as a second accelerant as telehealth expansion, online test result delivery, and reduced in-person contact made the portal a primary channel for routine care nearly overnight [2,3]. However, access to electronic health information does not by itself translate into portal activation or meaningful use. Activation, the manual step of logging into the portal for the first time, is the gateway on which every downstream function depends, from viewing results to messaging clinicians.

Portal access more than doubled in the past decade, with approximately 3 in 5 individuals accessing their records by 2022, and growth has continued since [4]. However, disparities by race, educational attainment, and geography have persisted into the postpandemic period [2,5,6]. In a nationally representative comparison of 2019 and 2022, Shah and Fiala [7] found that those gains left disparities by income, educational attainment, and age intact or widened them, and across studies, older age, Black or Hispanic race and ethnicity, lower income and educational attainment, and non-English primary language each predicted lower activation [8-12].

These disparities extend beyond patient characteristics to health system behavior. Richwine et al [13] found that Black and Hispanic patients were less likely than White patients to be offered portal access by a clinician and, among those offered, less likely to access it, whereas clinician encouragement increased access by just over 20 percentage points. Activation also varies with setting and implementation: when 3 health systems opened visit notes through their portals, more than 9 in 10 patients at a rural health system read at least one note vs fewer than half at an urban safety net hospital [14,15]. Even in an integrated system that actively promoted enrollment, just over 80% of White older adults registered for the portal vs just over half of Black older adults [16].

This divide has particular relevance for neurology patients. McGinley et al [17] mapped wide geographic disparities in access to neurologists across the United States, and for neurology patients managing chronic conditions, portal access supported monitoring test results, adjusting medications, and maintaining communication with clinicians [18]. Portal engagement has been associated with improved medication adherence and glycemic control among adults with diabetes [19] and with favorable outcomes in general patient populations [20], although comparable evidence specific to neurology populations remains limited. Neurological conditions also compound the barriers that limit portal use: cognitive and speech impairments complicate communication, whereas mobility limitations and driving restrictions constrain travel to in-person care, heightening reliance on the portal between visits [18,21].

What is less clear is how these demographic factors interact with neighborhood context and whether the answer depends on how finely geography is measured. Prior geographic work in neurology has operated at the national level [17], whereas portal disparity research has relied on single-center data or national surveys that cannot distinguish neighborhoods within a city [6,7], identifying who is disadvantaged but not where the disadvantage concentrates. Washington, DC (officially the District of Columbia), provides a strategic setting for examining these questions. Two academic hospitals share an electronic health record and a standard enrollment workflow yet serve populations with markedly different demographic profiles, and the district’s 8 wards encompass dramatic socioeconomic variation, with median household income ranging from approximately US $51,000 to US $142,000 and bachelor’s degree attainment ranging from 29% to 87% [22-24]. A shared portal infrastructure serving neighborhoods at the extremes of the socioeconomic distribution makes it possible to disentangle individual demographics, neighborhood context, and broader structural factors as contributors to portal disparities.

The aim of this study was to quantify demographic, socioeconomic, and geographic determinants of patient portal activation among neurology patients across 4 nested geographic scales: the DC metropolitan catchment area; DC’s 8 wards; census tracts, which most closely approximate neighborhoods; and residential zip codes. We hypothesized that activation would track neighborhood socioeconomic resources at every scale. We further hypothesized that racial disparities would persist within neighborhoods even after accounting for those resources.

## Methods

### Study Design and Setting

#### Overview

This cross-sectional study was conducted at 2 academic medical centers sharing a common electronic health record (Oracle Cerner; Oracle Corporation) in the Washington, DC, metropolitan area. Hospital A is a university-affiliated academic medical center with affiliated outpatient sites, and hospital B is an academic tertiary care hospital. Both hospitals operate under the health system’s standard institutional clinical visit workflow, including identical after-visit summary portal enrollment practices. This report follows the STROBE (Strengthening the Reporting of Observational Studies in Epidemiology) recommendations for cross-sectional studies (Checklist 1). Patients were eligible if they had at least one outpatient neurology encounter (including telehealth visits) at either center between February 25, 2021, and February 23, 2026; data were extracted on February 24, 2026, identifying 72,524 patients, of whom 107 (0.1%) were excluded for missing or indeterminate sex, a covariate in all adjusted models, yielding an analytic cohort of 72,417 (99.9%).

#### Sample Size, Power, and Precision

No sampling was performed; the cohort comprised the complete population of eligible patients identified from the electronic health record during the study window. Sample size was determined by the number of eligible patients rather than by an a priori power calculation. The analytic cohort of 72,417 patients provided high precision for individual-level estimates, and 95% CIs are reported throughout. Precision is necessarily lower for ecological analyses involving few geographic units (eg, 8 wards), and exact *P* values are reported for these analyses.

#### Measures and Covariates

Patient demographics, portal activation status, and residential addresses were extracted from the electronic health record. The primary outcome was patient portal activation status (active vs inactive) recorded at the time of data extraction. A patient was classified as active if they had logged into the patient portal at least once; activation requires a manual step by the patient. Covariates included age at the most recent encounter, sex, race and ethnicity, visit counts at each hospital, and year of the most recent encounter. Race and ethnicity were classified into 5 mutually exclusive categories: non-Hispanic White, non-Hispanic Black, Hispanic, non-Hispanic Asian, and other or unknown. Patients with any Hispanic ethnicity designation were classified as Hispanic regardless of race, consistent with how this field is captured in the electronic health record.

#### Geographic Assignment

Most patients resided in 1 of 3 jurisdictions (Maryland, DC, and Virginia), with the remainder residing in other states. The regional cohort analyzed in this study comprised all DC, Maryland, and Virginia residents, approximating the medical centers’ catchment area; this cohort was defined by state of residence rather than by distance from the hospitals, and residents of other states were excluded. For DC residents, patients were assigned to 1 of 8 wards based on their residential zip code and street address quadrant (northwest, northeast, southeast, and southwest). For addresses in multi-ward zip codes lacking a quadrant designator, street name matching was used as a secondary assignment method.

For the broader catchment area analysis, patient activation rates were aggregated by 5-digit zip code using 2 thresholds that served distinct purposes. A higher threshold (≥30 patients) for ecological correlation analyses ensured stable rate estimates suitable for statistical comparison, whereas suppression of cells with fewer than 10 patients in choropleth maps protected patient privacy in small geographic units. Additionally, patient street addresses were geocoded to census tracts using the US Census Bureau batch geocoder (“Public_AR_Current” benchmark), excluding post office boxes, homeless shelter addresses, and non-US postal codes.

#### Neighborhood Socioeconomic Variables

Neighborhood-level characteristics were obtained from the American Community Survey (ACS) 5-year estimates (2019-2023) via the *tidycensus* R package (version 1.6; K Walker) at the census tract level for DC, Maryland, and Virginia (3879 tracts). Three variables were selected: median household income, educational attainment (percentage with bachelor’s degree or higher), and broadband internet subscription rate. For ward-level analyses, ACS estimates were obtained directly at the ward level as DC wards are classified as state legislative districts (upper chamber) in census geography. For individual-level models, ACS variables were linked directly to each patient’s geocoded census tract. Patient addresses reflected the most recent address recorded in the health system; for patients with more than one address on file, the most recent one was used.

### Data Cleaning and Quality Assurance

Residential zip codes were standardized to 5-digit format, and 17 non-US postal codes were excluded from geographic analyses. Street addresses were parsed for quadrant designators (northwest, northeast, southeast, and southwest) to improve ward assignment in multi-ward zip codes. Patients with post office box addresses (664/72,417, 0.9%) were retained in demographic analyses but excluded from geographic mapping, and a small number of addresses listing homeless shelters (17/72,417, 0%) were similarly handled. Race and ethnicity fields were harmonized from multiple coding schemes in the electronic health record into 5 consistent categories.

### Statistical Analysis

Multivariable logistic regression estimated the association between patient characteristics and portal activation across all 3 cohorts: all patients, the regional cohort (DC, Maryland, and Virginia), and DC residents. Predictors included age (defined as age at most recent encounter), sex, race and ethnicity, visit counts at each hospital, and year of most recent encounter. Continuous variables were standardized to per-SD units, and the year of each patient’s last clinic visit was included to adjust for how recently they were seen. All model variables were complete in the analytic cohort, so no imputation was performed; incomplete geographic linkage affected only the tract-level analyses.

Geographic disparities were examined at 4 scales: ward-level and census tract–level correlations (Pearson and Spearman), zip code–level correlations, and individual-level neighborhood regression among DC residents. For individual-level regression, tract-level ACS data were linked directly to each patient’s geocoded census tract. Within-ward racial disparities were examined by stratifying activation by race and ethnicity applying the same 30-patient minimum to suppress unstable subgroups. Because the 3 neighborhood indicators measure overlapping dimensions of socioeconomic resources and were correlated, they were entered into separate models rather than combined; as a sensitivity analysis, elastic net regularization (L1+L2 penalty and 10-fold cross-validation) entered all 3 simultaneously to identify which retained independent predictive value. Two additional sensitivity analyses refit the primary model, first restricted to patients whose most recent encounter occurred in 2024 to 2026 and, second, excluding patients in the “other or unknown” race and ethnicity category.

### Geospatial Visualization

Choropleth maps were generated using *sf* and *ggplot2* in R. Regional maps display census tract–level data within an 8-mile square around central Washington, DC. Ward-level activation rates and ACS indicators are presented in tabular form. All analyses were conducted in R (version 4.3; R Foundation for Statistical Computing), with census tract geometries obtained via the *tigris* package (version 2.0; K Walker).

### Ethical Considerations

This study was reviewed and approved by the MedStar Health Research Institute Institutional Review Board (STUDY00007139) on October 19, 2023. This study was determined to be exempt (category 4[iii] [25]: secondary research on data or specimens), and the requirements for informed consent and HIPAA (Health Insurance Portability and Accountability Act) authorization were waived. All data were deidentified prior to analysis. No compensation was provided as this was a secondary analysis of existing records and patients were not contacted. No individual participants are identifiable in the manuscript, tables, or figures.

## Results

### Study Population

**Figure 1.**
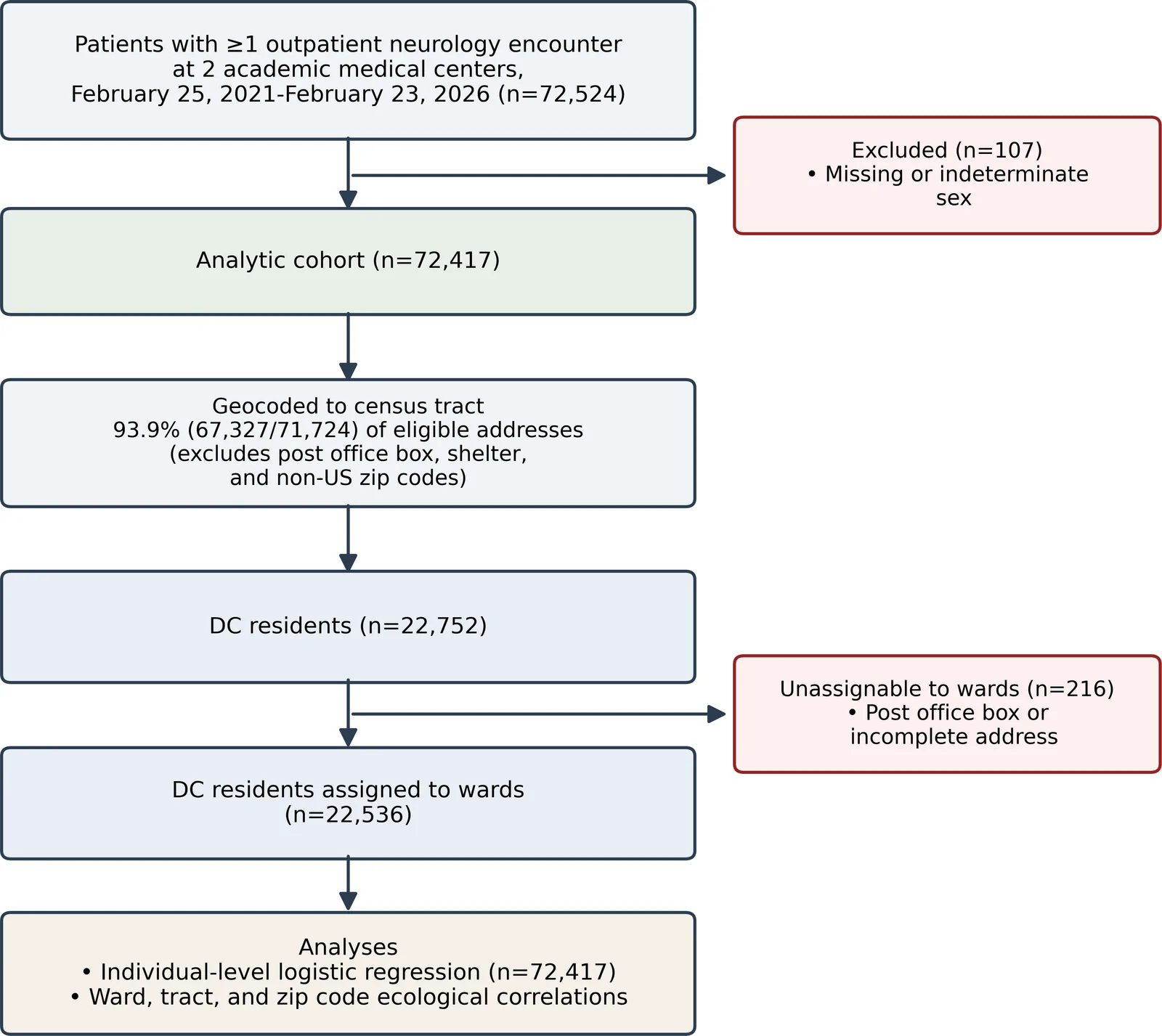
Flow of patients through identification, exclusion, geocoding, and ward assignment in this cross-sectional study of neurology patients in the Washington, DC, metropolitan area (February 2021-February 2026).

Table 1 presents the study population across 3 nested cohorts (Figure 1). Among all 72,417 patients, the mean age was 56.0 (SD 18.8) years, 62.4% (n=45,182) were female, and 38.9% (n=28,154) were non-Hispanic White individuals. Most patients (55,330/72,417, 76.4%) were seen at hospital A and affiliated sites only, with 20.1% (14,525/72,417) seen at hospital B only and 3.5% (2562/72,417) seen at both sites. Overall portal activation was 64.7% (46,851/72,417). Activation was higher among patients seen at both sites (1956/2562, 76.3%) than at hospital A only (37,248/55,330, 67.3%) or hospital B only (7647/14,525, 52.6%). Of 22,752 DC residents, 22,536 (99.1%) were assigned to a ward, and that assigned group is the denominator for the three assignment methods. Within those 22,536, a total of 95.2% (n=21,446) were assigned by residential zip code plus quadrant, 4.4% (n=990) by unambiguous zip code alone, and 0.4% (n=100) by street-level name matching. Geocoding matched 93.9% (67,327/71,724) of eligible addresses to a census tract, including 94.8% (21,567/22,752) of DC residents across 206 tracts, and geocoded and nongeocoded patients were demographically similar (Table S1 in Multimedia Appendix 1). For the broader catchment analysis, activation was aggregated across 130 zip codes (51,506/72,417, 71.1% of the patients).

**Table 1.** Study population characteristics across 3 cohorts. The regional cohort comprises DC, Maryland (MD), and Virginia (VA) residents.

Characteristic	All patients	Regional (DC, MD, and VA; n=69,071)	DC residents (n=22,752)
Portal activated, n (%)	46,851 (64.7)	44,568 (64.5)	14,503 (63.7)
Age (y), mean (SD)	56.0 (18.8)	56.2 (18.8)	53.4 (18.6)
Female sex, n (%)	45,182 (62.4)	43,119 (62.4)	14,772 (64.9)
Visits per patient, mean (SD)	9.7 (18.9)	9.7 (18.9)	7.9 (14.3)
Race and ethnicity, n (%)			
Hispanic	3600 (5)	3485 (5)	1675 (7.4)
Non-Hispanic Asian	1925 (2.7)	1858 (2.7)	273 (1.2)
Non-Hispanic Black	22,900 (31.6)	22,540 (32.6)	10,744 (47.2)
Non-Hispanic White	28,154 (38.9)	26,299 (38.1)	6594 (29)
Other or unknown	15,838 (21.9)	14,889 (21.6)	3466 (15.2)
Hospital site, n (%)			
Hospital A only	55,330 (76.4)	52,400 (75.9)	11,807 (51.9)
Hospital B only	14,525 (20.1)	14,193 (20.5)	9542 (41.9)
Both sites	2562 (3.5)	2478 (3.6)	1403 (6.2)
Jurisdiction, n (%)			
DC	22,752 (31.4)	22,752 (32.9)	—^a^
Maryland	28,303 (39.1)	28,303 (41)	—
Virginia	18,016 (24.9)	18,016 (26.1)	—
Other	3346 (4.6)	—	—
Most recent encounter year, n (%)			
2021	6619 (9.1)	6243 (9)	2279 (10)
2022	9398 (13)	8896 (12.9)	3211 (14.1)
2023	11,961 (16.5)	11,291 (16.3)	3803 (16.7)
2024	13,076 (18.1)	12,369 (17.9)	4106 (18)
2025	23,151 (32)	22,267 (32.2)	7090 (31.2)
2026	8212 (11.3)	8005 (11.6)	2263 (9.9)

a Not applicable.

### Activation Rates by Subgroup

Activation rates varied markedly by race and ethnicity across all scales (Table 2). Non-Hispanic White patients activated at 76.1% (21,420/28,154) overall compared to 57% (13,057/22,900) for non-Hispanic Black patients, 57.6% (1109/1925) for non-Hispanic Asian patients, and 55% (1979/3600) for Hispanic patients. These disparities were amplified within DC, with non-Hispanic White patients at 87.1% (5744/6594), non-Hispanic Black patients at 51.1% (5488/10,744), and Hispanic patients at 44% (737/1675), whereas non-Hispanic Asian patients in DC activated at 82.1% (224/273). Hospital A achieved higher activation than hospital B in all cohorts (37,248/55,330, 67.3% vs 7647/14,525, 52.6% overall), reflecting differences in patient demographics rather than portal infrastructure.

**Table 2.** Portal activation rates by patient subgroup across 3 cohorts.

Subgroup	All patients, n/N (%)	Regional (DC, MD^a^, and VA^b^), n/N (%)	DC residents, n/N (%)
Overall	46,851/72,417 (64.7)	44,568/69,071 (64.5)	14,503/22,752 (63.7)
Race and ethnicity			
Hispanic	1979/3600 (55)	1888/3485 (54.2)	737/1675 (44)
Non-Hispanic Asian	1109/1925 (57.6)	1061/1858 (57.1)	224/273 (82.1)
Non-Hispanic Black	13,057/22,900 (57)	12,835/22,540 (56.9)	5488/10,744 (51.1)
Non-Hispanic White	21,420/28,154 (76.1)	20,015/26,299 (76.1)	5744/6594 (87.1)
Other or unknown	9286/15,838 (58.6)	8769/14,889 (58.9)	2310/3466 (66.6)
Hospital site			
Hospital A only	37,248/55,330 (67.3)	35,295/52,400 (67.4)	9034/11,807 (76.5)
Hospital B only	7647/14,525 (52.6)	7393/14,193 (52.1)	4485/9542 (47)
Both sites	1956/2562 (76.3)	1880/2478 (75.9)	984/1403 (70.1)

a MD: Maryland.

b VA: Virginia.

### Individual-Level Predictors of Portal Activation

Multivariable logistic regression revealed consistent predictors across all 3 cohorts (Table 3). Age was the strongest predictor. Older patients activated at substantially lower rates, with each SD increase in age associated with 37% to 40% lower odds of activation. Racial and ethnic disparities were amplified from the full cohort to the DC cohort. The non-Hispanic Black patient adjusted odds ratio (aOR) was 0.46 (95% CI 0.44-0.48) overall vs 0.19 (95% CI 0.17-0.20) in DC, the Hispanic patient aOR was 0.34 (95% CI 0.31-0.37) overall vs 0.11 (95% CI 0.10-0.13) in DC, and the non-Hispanic Asian patient aOR was 0.47 (95% CI 0.42-0.52) overall vs 0.66 (95% CI 0.47-0.91) in DC. Hospital A visit frequency was strongly associated with activation across all scales, with models adjusting for the year of each patient’s last clinic visit.

**Table 3.** Multivariable logistic regression: predictors of portal activation across 3 cohorts^a^.

Predictor	All patients, aOR^b^ (95% CI)	Regional (DC, MD^c^, and VA^d^), aOR (95% CI)	DC residents, aOR (95% CI)
Age (per SD)	0.60 (0.59‐0.61)	0.60 (0.59‐0.62)	0.63 (0.61‐0.65)
Male sex (reference: female)	0.70 (0.67‐0.72)	0.70 (0.68‐0.73)	0.65 (0.61‐0.69)
NHB^e^ patients (reference: NHW^f^ patients)	0.46 (0.44‐0.48)	0.46 (0.44‐0.48)	0.19 (0.17‐0.20)
Hispanic patients (reference: NHW patients)	0.34 (0.31‐0.37)	0.33 (0.30‐0.35)	0.11 (0.10‐0.13)
NHA^g^ patients (reference: NHW patients)	0.47 (0.42‐0.52)	0.46 (0.41‐0.51)	0.66 (0.47‐0.91)
Patients of other or unknown races (reference: NHW patients)	0.42 (0.40‐0.44)	0.42 (0.40‐0.44)	0.28 (0.25‐0.31)
Hospital A visits (per SD)	3.24 (3.08‐3.41)	3.15 (2.99‐3.31)	3.58 (3.16‐4.04)
Hospital B visits (per SD)	1.14 (1.12‐1.16)	1.13 (1.10‐1.15)	1.03 (1.00‐1.05)
Year of last patient encounter (per SD)	1.17 (1.15‐1.19)	1.18 (1.16‐1.20)	1.21 (1.17‐1.25)

a All models adjusted for the listed variables simultaneously. Continuous variables standardized (per SD). *P*<.001 in all cases except for hospital B visits in DC (*P*=.049).

b aOR: adjusted odds ratio.

c MD: Maryland.

d VA: Virginia.

e NHB: non-Hispanic Black.

f NHW: non-Hispanic White.

g NHA: non-Hispanic Asian.

### Geographic Variation by DC Ward

**Figure 2.**
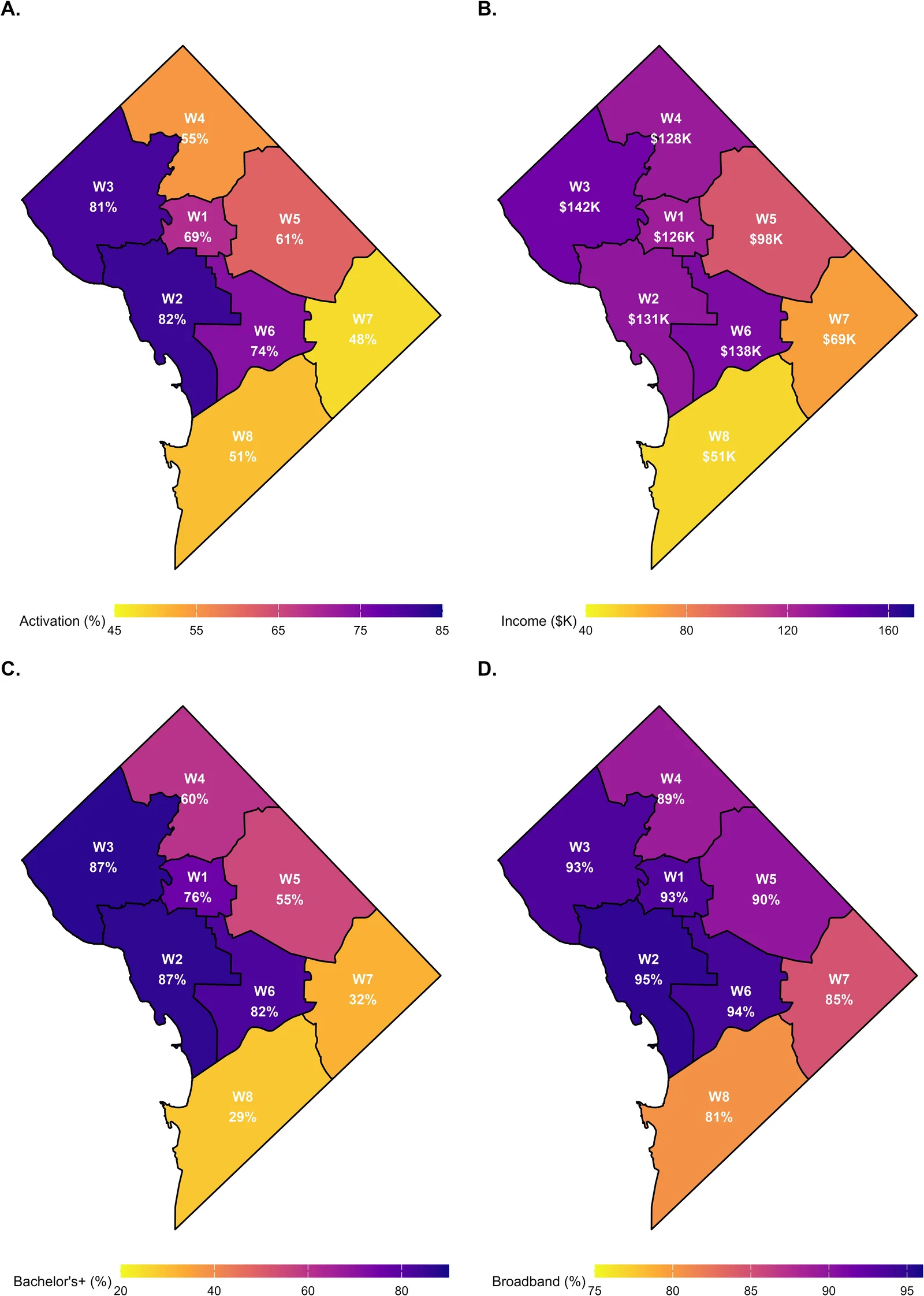
DC ward–level portal activation and neighborhood characteristics. Choropleth maps of the 8 DC wards showing (A) portal activation rate, (B) median household income, (C) bachelor’s degree attainment or higher, and (D) broadband internet access (n=22,536 DC patients; neighborhood characteristics were obtained from American Community Survey [ACS] 5-year estimates [2019-2023]).

**Figure 3.**
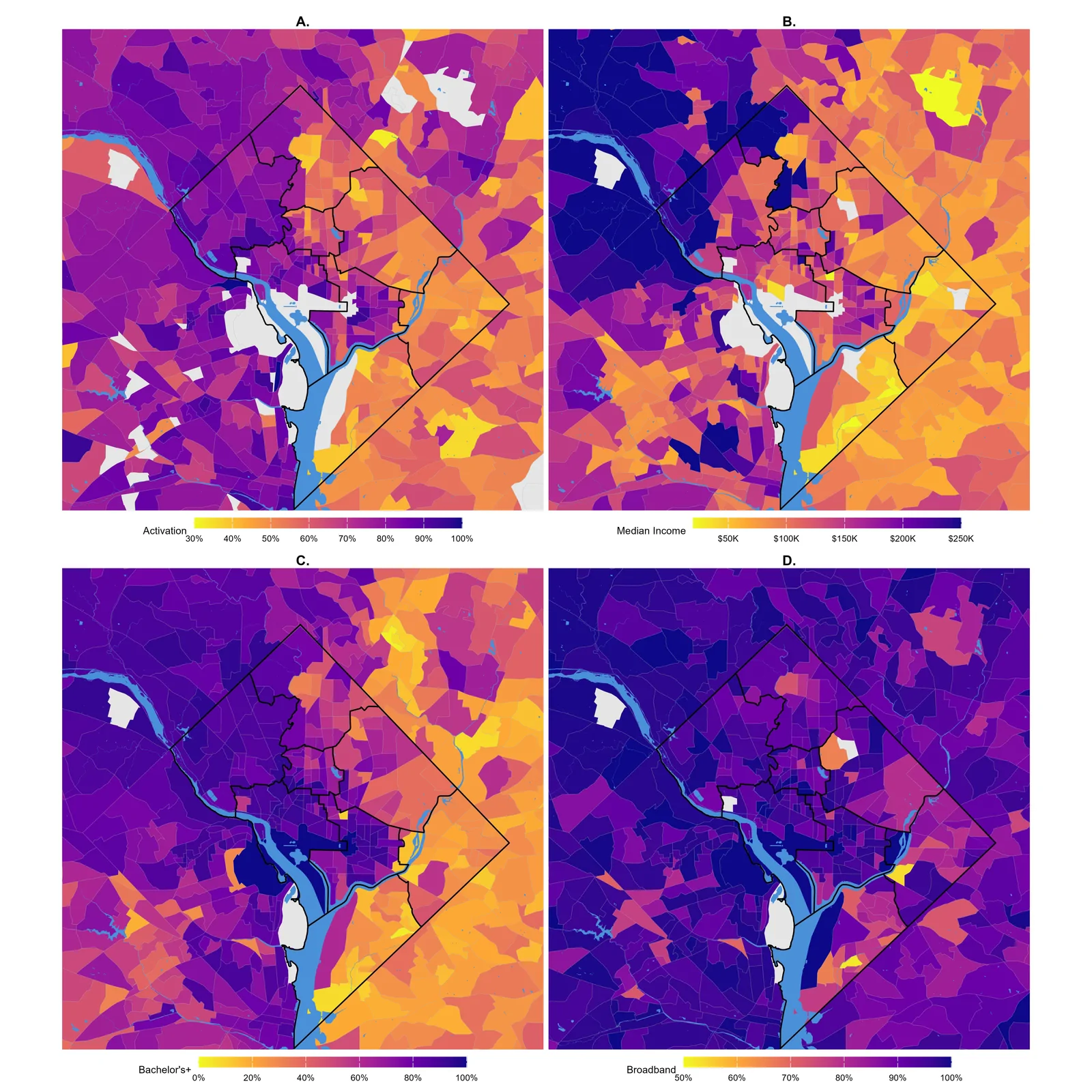
Regional geographic distribution of portal activation and neighborhood characteristics. Census tract–level choropleth maps of an 8-mile square around central Washington, DC. Panel A shows patient portal activation rate (tracts with fewer than 10 patients were suppressed); panels B to D show American Community Survey (ACS) 5-year estimates (2019-2023) for median household income, educational attainment, and broadband access. DC ward boundaries are overlaid; water bodies and tracts without data are unshaded.

Among 22,536 DC residents assigned to wards, activation rates ranged from 48% (1023/2131) in ward 7 to 82% (1554/1896) in ward 2, a 34.0–percentage point gap (Table 4; Table S2 in Multimedia Appendix 1). The highest-activation wards (2, 3, and 6) had rates of 74.3% (1408/1895) to 82% (1554/1896), whereas wards east of the Anacostia River (wards 7 and 8) had rates below 51% (Figure 2). This geographic pattern is visually striking across the regional maps. Portal activation, median income, educational attainment, and broadband access all declined along a west-to-east gradient across the metropolitan area, with the sharpest transitions occurring at the Anacostia River (Figure 3).

**Table 4.** Portal activation and neighborhood characteristics by DC ward^a^.

Ward	Patients, n	Activation, n/N (%)	Median income (US $)	Bachelor’s degree (%)	Broadband access (%)
1	2985	2056/2985 (68.9)	126,000	76	93
2	1896	1554/1896 (82)	131,000	87	95
3	2831	2285/2831 (80.7)	142,000	87	93
4	4264	2341/4264 (54.9)	128,000	60	89
5	3874	2367/3874 (61.1)	98,000	55	90
6	1895	1408/1895 (74.3)	138,000	82	94
7	2131	1023/2131 (48)	69,000	32	85
8	2660	1351/2660 (50.8)	51,000	29	81

a Activation rates are calculated among DC residents assigned to each ward (22,536/72,417, 31.1% of the patients). Neighborhood characteristics are American Community Survey 5-year estimates (2019-2023) obtained at the ward level (state legislative district, upper chamber). Ward 7 had the lowest activation (1023/2131, 48%), and ward 2 had the highest (1554/1896, 82%).

### Ecological Correlations

Ward-level correlations were strong and significant (Table 4; educational attainment: *r*=0.95 and *P*<.001; broadband access: *r*=0.89 and *P*=.003; median income: *r*=0.81 and *P*=.01). Census tract–level correlations among 201 DC tracts with 30 or more patients confirmed these patterns (educational attainment: *r*=0.85 and *P*<.001; income: *r*=0.66 and *P*<.001; broadband access: *r*=0.60 and *P*<.001). Among 698 tracts across the full regional catchment, correlations were attenuated but remained significant (educational attainment: *r*=0.42; income: *r*=0.25; broadband access: *r*=0.22; *P*<.001 in all cases). Zip code–level correlations were weakest (Table S3 in Multimedia Appendix 1). Spearman correlations (ρ) confirmed these patterns at all scales.

### Within-Ward Racial Disparities

Racial disparities persisted within individual wards. In ward 6 (median annual income of US $138,000; 82% with a bachelor’s degree), non-Hispanic White patients activated at 91.2% (714/783) vs 53.6% (381/711) for non-Hispanic Black patients, a 37.6-point gap. Across wards 1 to 6, non-Hispanic White patient activation ranged from 85.7% (1551/1809) to 91.2% (714/783), whereas non-Hispanic Black patient activation ranged from 50.5% (1139/2255) to 63.6% (206/324). This demonstrates that neighborhood resources alone do not explain disparities.

### Neighborhood-Level Predictors

Among DC residents (22,536/72,417, 31.1%), each ward-level socioeconomic variable was significantly associated with activation after demographic adjustment (Table S4 in Multimedia Appendix 1). Per SD, the aORs were 1.18 (95% CI 1.14‐1.21) for income, 1.26 (95% CI 1.22‐1.30) for educational attainment, and 1.22 (95% CI 1.18‐1.26) for broadband access. When tract-level ACS data were linked directly to geocoded DC patients (n=21,567; adjusted models: n=21,335), the associations were consistently stronger, with aORs per SD of 1.31 (95% CI 1.26‐1.35) for income, 1.37 (95% CI 1.32‐1.42) for educational attainment, and 1.22 (95% CI 1.18‐1.26) for broadband access. Attenuation from unadjusted to adjusted estimates reflects overlap between neighborhood characteristics and patient race and ethnicity. In a joint model entering all 3 indicators simultaneously, the independent association with broadband access attenuated to the null (aOR 0.99, 95% CI 0.95-1.03), whereas educational attainment remained the strongest predictor, indicating that the univariate association with broadband access is largely explained by its overlap with income and educational attainment (Table S5 in Multimedia Appendix 1).

### Sensitivity Analyses

The year of the last clinic visit was included as a control rather than as a measure of secular trend; because it reflects only the timing of that visit rather than longitudinal observation, it cannot distinguish improving adoption from selective retention of engaged patients (Table S6 in Multimedia Appendix 1). To verify that the observed racial disparities were not an artifact of this temporal structure, we conducted a sensitivity analysis restricted to patients whose most recent encounter occurred in 2024 to 2026 (44,439/72,417, 61.4%). Disparities persisted essentially unchanged. Relative to non-Hispanic White patients, adjusted odds of activation were 0.37 (95% CI 0.35‐0.39) for non-Hispanic Black patients, 0.28 (95% CI 0.26‐0.31) for Hispanic patients, and 0.39 (95% CI 0.34‐0.44) for non-Hispanic Asian patients (*P*<.001 in all cases), consistent with disparities being independent of temporal confounding. To assess whether the large “other or unknown” category influenced the estimated disparities, we also refit the primary model excluding the 21.9% (15,838/72,417) of the patients of other or unknown race and ethnicity (n=56,579). Adjusted estimates were unchanged: relative to non-Hispanic White patients, odds of activation were 0.46 (95% CI 0.44‐0.48) for non-Hispanic Black patients, 0.34 (95% CI 0.31‐0.36) for Hispanic patients, and 0.47 (95% CI 0.42‐0.52) for non-Hispanic Asian patients (*P*<.001), indicating that classification into this group does not drive the reported disparities.

### Facility Care Setting Analysis

In an exploratory analysis, we identified patients whose most recent recorded address contained a care facility name (203/72,417, 0.3% of the cohort). Activation among these patients was far below the 64.7% (46,851/72,417) cohort activation at 19.5% (15/77) in nursing or skilled nursing facilities, 16.9% (11/65) in group homes, and 20.2% (41/203) across all facility care settings combined. These patients were too few to materially affect the overall estimates.

## Discussion

### Principal Findings

Three findings carry the weight of this analysis: among neighborhood characteristics, educational attainment was the strongest predictor of activation; racial disparities persisted within even the most affluent wards; and among individual characteristics, age was the strongest predictor of nonactivation. Consistent with our hypotheses, portal activation tracked neighborhood socioeconomic resources at every geographic scale examined. Educational attainment was a stronger predictor than income, whose association with activation diminished after adjustment for age, sex, and race and ethnicity, suggesting that education captures aspects of digital readiness that income alone does not [6]. The more consequential finding is what neighborhood resources failed to do: substantial racial disparities in activation persisted within individual wards, including the most affluent, even after accounting for socioeconomic resources. Age was the strongest individual predictor, but it was the combination of race and place, not any single factor, that defined the divide.

The 21st Century Cures Act made real-time electronic access the default standard of care in April 2021 [1], and the pandemic had already moved routine communication and results to digital channels, as Nishii et al documented [3,^26^]. Our observation window, February 2021 to February 2026, covers the 21st Century Cures Act’s implementation and the years following the pandemic’s shift to digital care. The disparities we report are therefore those that persisted once electronic access had become the norm, consistent with national data that recent gains did not narrow racial and ethnic disparities [2].

### Comparison With Prior Work

Across prior work, disparities were established nationally or at a single geographic scale. By geocoding patients to census tracts and comparing nested scales within one metropolitan area, this study localized them. To our knowledge, this is the first multi-scale geographic analysis of patient portal activation. The strength of the association varied with the scale: it was strongest across DC’s 8 wards and weaker at the finer census tract level and the broader regional (DC, Maryland, and Virginia) and zip code samples.

At the neighborhood level, Perzynski et al [8] linked lower portal use to broadband inequality among general outpatients in a single county using broadband as the sole neighborhood measure at one geographic scale. The present study extends this approach to a neurology population across an entire metropolitan area, comparing 4 nested geographic scales and 3 socioeconomic indicators. The broadband association was replicated at the ward and tract levels, and neighborhood educational attainment was consistently the stronger correlate. At the individual level, Sarkar et al [10] documented lower portal use among Black and Hispanic patients with diabetes but did not link those disparities to where patients lived. Those gaps are replicated in this cohort. Such findings identify who is disadvantaged without revealing whether neighborhood resources mitigate or magnify that disadvantage.

At the population level, Shah and Fiala [7] showed that national gains in portal access and use up to 2022 left disparities by income, educational attainment, and age intact or widened them, and a meta-analysis by Goldberg et al [6] confirmed that socioeconomic and demographic factors drive the digital divide; both, however, aggregated national surveys or pooled findings across studies rather than resolving variation within a single community [6,7]. The national surveys by Richwine [2] likewise documented persistent racial and ethnic disparities in access to electronic health information. Such national estimates treat each group as a fixed block; our data instead show the gap concentrating by race even within a single ward and after adjustment for age, a pattern more consistent with structural and social factors than with disease. This mirrors the 27-point racial registration gap observed within a single system that promoted enrollment to all members [16]. Plausible mechanisms include differences in how enrollment is offered [13], lower institutional trust shaped by concerns that demographic information may be used to discriminate [27], and unmeasured gaps in digital literacy and device access [6], although our data cannot distinguish among them. This aligns with DC’s documented health inequities, where differences in life expectancy span as much as 21 years across neighborhoods [23].

In that same integrated system, older adults used patient portals and other eHealth tools less than younger members [16], and Anthony et al [12] identified similar age gradients in a national sample. The same gradient dominated here: age was the strongest individual predictor of nonactivation. This likely reflects both lower technological literacy and generational differences in how patients expect to interact with the health system [12,16], a distinction that grows as care moves toward digital-first communication.

Viewed through the 3-level model of the digital divide by van Deursen and Helsper [28], which distinguishes access to digital tools (first level), their use (second level), and the proficiency to benefit from them (third level), our findings locate the disparities at the second level. Activation gaps persisted in the wards where first-level resources such as broadband access and income are most abundant, and neighborhood educational attainment, a marker of digital skills rather than of access, was the dominant area-level predictor. The third-level question, whether nonactivation translates into worse clinical outcomes, is the one our data motivate but cannot answer.

### Practical Implications for Health Systems

For neurology patients managing complex chronic conditions, portal access supports medication management, appointment coordination, and result review [21], functions for which access remains unequally distributed. To equalize that access, these findings point to several actionable measures. First, digital literacy support, including in-clinic enrollment assistance and community health worker outreach, would let systems concentrate limited resources in the geographies with the lowest activation, which the ward- and tract-level maps presented here identify directly [29,30]. Targeting can begin without patient-level geocoding: the ward-level analyses identified the same west-to-east gradient as tract-level linkage, so systems can start with whatever administrative geography is available (wards here, council districts, zip codes, or census tracts elsewhere) and refine where street-level precision is needed. Second, in diverse regions such as Washington, DC, multilingual onboarding and interpreter-integrated portal support can address language-related barriers [31,32]. Third, although a clinician’s primary responsibility is direct patient care, national data show that patients whose clinicians offer and encourage enrollment are substantially more likely to activate [13]; brief clinician education can be paired with adequate enrollment staff so that the burden does not fall on clinicians alone. Finally, converting enrollment from opt in to opt out is feasible and effective: SMS text message autoenrollment at an academic health system tripled activation among Black, Hispanic, and Asian patients and narrowed racial and language-based gaps without eliminating them [33].

Activation itself can also serve as an equity metric: stratifying rates by race, ethnicity, and neighborhood using data already captured in the electronic health record would make gaps such as these visible in routine quality reporting. These measures carry 2 caveats. Many patients, particularly older adults and those from minoritized groups, prefer direct conversation to secure messaging [13,16], and rising portal volume adds uncompensated clinician workload. The goal is therefore a tool that, integrated thoughtfully, extends rather than replaces the therapeutic relationship.

As medicine becomes increasingly data driven, these findings also carry weight for research. The parameters that structured activation in this cohort (age, race and ethnicity, educational attainment, and neighborhood) are the same inputs that informatics models require, and prior work has shown that they are unevenly captured in the record: more than a fifth of this cohort (15,838/72,417, 21.9%) lacked informative race and ethnicity data, and in prior studies, recorded values frequently disagreed with what patients themselves reported [27,34]. Data collected through the portal narrow the lens further because they exist only for patients who activated, so studies built on patient-reported outcomes, messages, or remote monitoring will underrepresent older patients, Black and Hispanic patients, and residents of lower-resourced neighborhoods, echoing the underrepresentation that limits the applicability of clinical trial findings [35]. This is a problem of data integrity as much as access: the record serves as a virtual twin of the patient population; models are only as faithful as the twin; and measuring and reporting these parameters, including activation status, is a prerequisite for representative digital health research.

### Limitations

Among the study’s measurement constraints, portal activation was captured as a single binary status without the date of enrollment, so we could not measure adoption over time. The year variable likewise reflects each patient’s last clinic visit rather than longitudinal follow-up, so we cannot establish temporal trends or disentangle the contributions of the 21st Century Cures Act and pandemic-era changes. Broadband subscription also does not reflect internet quality, digital literacy, or device access.

Two further limitations bear on interpretation. The ecological correlations are subject to the ecological fallacy [36], although the associations held at the finer tract and zip code levels, where aggregation bias is smaller. The 2 sites also belong to a single health system, which limits generalizability. The large “other or unknown” race and ethnicity category also warrants attention. If patients in this category differ systematically from those with recorded race and ethnicity, it could mask additional disparities or dilute those we report. A sensitivity analysis excluding this group left the adjusted estimates unchanged, but the reported gaps are best interpreted as estimates among patients with recorded race and ethnicity.

### Conclusions

Disparities in patient portal activation among neurology patients are multifactorial; age, race, neighborhood context, and health system factors each contribute independently but compound in combination. Within a single city, the result is 2 populations: patients for whom the portal is routine and patients for whom it is out of reach. Because this gap accumulates across structural, demographic, and disease-related barriers, no single intervention can close it. Infrastructure investment alone cannot overcome low digital literacy, and culturally tailored outreach cannot compensate for neighborhoods without reliable internet connectivity; closing the divide requires coordinated efforts aimed at the populations and places where it is widest.

Several questions remain for neurology specifically: whether activation gaps widen among patients with cognitive or motor impairment and what tailored interventions, such as simplified portal interfaces for Parkinson disease or dementia, can offer. Individual-level prediction of patients at highest risk of nonactivation incorporating insurance status, primary language, and social vulnerability is a further priority, as is whether nonactivation itself marks patients at risk of worse clinical outcomes, a question requiring linkage to diagnosis, treatment, and survival data beyond the present dataset.

This analysis maps where the digital divide in health care runs deepest, providing a foundation for targeted intervention. As portals become the default channel for results, scheduling, and communication, activation is no longer a convenience but a precondition for full participation in care, and distributing it equitably is a patient safety and health equity obligation. Patients who are not digitally engaged may find their care affected not by its availability but by the expanding digital toolkit reshaping the practice of medicine.

## Supplementary material

10.2196/98695 Multimedia Appendix 1 Geocoding comparison, ward-level American Community Survey indicators, zip code-level correlations, ward-level neighborhood regression models, joint neighborhood-indicator models, and temporal sensitivity analyses.

10.2196/98695 Checklist 1 STROBE checklist.

## Abbreviations

ACS: American Community Survey
aOR: adjusted odds ratio
DC: District of Columbia
HIPAA: Health Insurance Portability and Accountability Act
STROBE: Strengthening the Reporting of Observational Studies in Epidemiology
