# Supplementary material for: Patient Portal Activation Disparities by Ward, Census Tract, and Zip Code in an Urban Landscape Among Neurology Patients: Cross-Sectional Multi-Scale Analysis
Source: J Med Internet Res. 2026 Aug 26;28:e98695. doi: 10.2196/98695 (PMC13515356; doi:10.2196/98695)
Supplement: Supplementary file 2 [file jmir-v28-e98695-s002.docx]

# STROBE Statement — Checklist of Items for Cross-Sectional Studies

**Manuscript #98695:** Patient Portal Activation Among Neurology Patients: A Multiscale Analysis of Disparities by Ward, Census Tract, and ZIP Code in Washington, DC

| Item | No. | Recommendation | Location in manuscript |
| --- | --- | --- | --- |
| Title and abstract | 1 | (a) Indicate the study’s design in the title or abstract; (b) provide an informative and balanced summary | Abstract (Methods: “cross-sectional study”; structured abstract) |
| Background/rationale | 2 | Explain the scientific background and rationale | Introduction, paragraphs 1–4 |
| Objectives | 3 | State specific objectives, including any prespecified hypotheses | Introduction, final paragraph (aims and hypotheses) |
| Study design | 4 | Present key elements of study design early in the paper | Methods, Study Design and Setting |
| Setting | 5 | Describe the setting, locations, and relevant dates | Methods, Study Design and Setting (February 2021–February 2026) |
| Participants | 6 | Give eligibility criteria and sources/methods of selection | Methods, Study Design and Setting; Figure 3 |
| Variables | 7 | Define all outcomes, exposures, predictors, and effect modifiers | Methods, Outcome Definition; Demographic Variables; Geographic Assignment; Neighborhood Measures |
| Data sources/measurement | 8 | Give sources of data and details of methods of assessment | Methods, Data Sources (EHR); Geographic Assignment (US Census Bureau geocoder); Neighborhood Measures (American Community Survey) |
| Bias | 9 | Describe any efforts to address potential sources of bias | Methods, Sensitivity Analyses; Supplementary Table S1 (geocoded vs non-geocoded); Discussion, Limitations |
| Study size | 10 | Explain how the study size was arrived at | Methods, Study Design and Setting (all eligible patients; N = 72,417); Figure 3 |
| Quantitative variables | 11 | Explain how quantitative variables were handled | Methods, Statistical Analysis (standardization of age and visit counts; encounter year as recency control) |
| Statistical methods | 12 | (a) Describe all statistical methods, including confounding control; (b) subgroups and interactions; (c) missing data; (d) analytical methods accounting for sampling strategy; (e) sensitivity analyses | Methods, Statistical Analysis (multivariable logistic regression; ward-stratified analyses; exclusion for missing sex; ecological correlations at each scale; two sensitivity analyses) |
| Participants (results) | 13 | Report numbers at each stage; reasons for non-participation; flow diagram | Results; Figure 3 |
| Descriptive data | 14 | Characteristics of participants; missing data for each variable | Results, Participant Characteristics; Table 1; Supplementary Table S1 |
| Outcome data | 15 | Report numbers of outcome events or summary measures | Results (activation 64.7%, 46,851/72,417; rates by group); Table 2 |
| Main results | 16 | Give unadjusted and adjusted estimates with precision; report category boundaries | Results, adjusted models (aORs with 95% CIs); Tables 2–4; Supplementary Table S4 |
| Other analyses | 17 | Report other analyses done (subgroups, interactions, sensitivity) | Results, ward-level, tract-level, and zip-level analyses; Sensitivity Analyses |
| Key results | 18 | Summarise key results with reference to study objectives | Discussion, Principal Findings |
| Limitations | 19 | Discuss limitations, sources of potential bias, direction and magnitude | Discussion, Limitations |
| Interpretation | 20 | Give a cautious overall interpretation considering objectives, limitations, and other evidence | Discussion, Comparison With Prior Work; Practical Implications |
| Generalisability | 21 | Discuss the generalisability (external validity) | Discussion, Limitations (single health system); regional (DC/MD/VA) cohort analyses |
| Funding | 22 | Give the source of funding and the role of the funders | Funding statement |
